# Human Placenta and Markers of Heavy Metals Exposure: Esteban-Vasallo et al. Respond

**DOI:** 10.1289/ehp.1206061R

**Published:** 2013-01-01

**Authors:** María D. Esteban-Vasallo, Nuria Aragonés, Marina Pollan, Gonzalo López-Abente, Beatriz Perez-Gomez

**Affiliations:** 1 Subdirectorate for Health Promotion and Prevention, Madrid Regional Health Authority, Madrid, Spain; 2 National Center for Epidemiology/CIBERESP, Carlos III Institute of Health, Madrid, Spain, E-mail: bperez@isciii.es

We appreciate the interest of Pigatto et al. in our review (Esteban-Vasallo et al. 2012). We understand their concern regarding mercury amalgams; however, the purpose of our review was to summarize the available information on total mercury, cadmium, and lead levels in human placental tissue, obtained from studies that reported original quantitative data. Published evidence suggests a possible association between mercury released from mercury-containing dental amalgam fillings and levels of this metal in diverse fetal tissues (kidney, brain, and cord blood) (Drasch et al. 1994). In contrast, studies focusing on human placenta and amalgams are scarce and their results inconsistent. The only two studies included in our review that assessed a possible relationship between dental fillings and total mercury—a small study in Taiwan (46 women) (Hsu et al. 2007) and another in Jamaica (52 women) (Grant et al. 2010)—found no association. Only Ask et al. (2002) reported higher mercury levels in mothers with a higher number of fillings, but they studied inorganic mercury and not total mercury.

None of the studies mentioned by Pigatto et al. in their letter (Clarkson and Magos 2006; Gundacker and Hengstschläger 2012; Richardson et al. 2011) includes original data, although we did identify an additional reference from those articles that might provide more data on this issue, a symposium abstract by Ursinyova et al. (2006). In this abstract, the authors described a significant correlation between the number of amalgams and placental mercury levels in 409 women; however, these findings have not yet been published in a full report that would allow us to better evaluate the results. In addition, Wannag and Skjaeråsen (1975) seemed to provide original information, but we were unable to find this paper for our review. In this context, we have to disagree with Pigatto et al.; in our opinion, the association between mercury exposure from dental amalgam fillings and levels of this metal in human placenta cannot yet be considered as well-established.
